# Editorial: Mitochondrial sensitivity of sensory neurons controlling breathing

**DOI:** 10.3389/fphys.2022.1068341

**Published:** 2022-10-28

**Authors:** Nicholas Jendzjowsky, Andrew M. Coney

**Affiliations:** ^1^ The Lundquist Institute for Biomedical Innovation at Harbor UCLA, Torrance, CA, United States; ^2^ Respiratory and Critical Care Medicine and Physiology, David Geffen School of Medicine UCLA, Los Angeles, CA, United States; ^3^ School of Biomedical Sciences, Institute of Clinical Sciences, The Medical School, University of Birmingham, Birmingham, United Kingdom

**Keywords:** mitochondia, control of breathing, oxygen sensitivity, hypoxia, fetal development

We welcome the reader to a special edition of Frontiers in Physiology with a focus on “Mitochondrial Sensitivity of Sensory Neurons Controlling Breathing”. This featured topic highlights one research article and three reviews which span mitochondrial oxygen sensing mechanisms, the paradoxical effect of hyperoxic brainstem toxicity and, mitochondrial dysfunction and ensuing sequelae during development. First, an up-to-date summary of the potential convergence of multiple mitochondrial-mediated oxygen-sensitive pathways are discussed in the context of specificity to stimulation parameters delivered to the carotid body and how the chemo-sensitive cells could orchestrate converging pathways to elicit specificity of oxygen sensing (Holmes et al.). Next, we explore a novel oxygen-sensing mechanism within the glomus cells of the carotid chemoreceptors surrounding mitochondrial heat generation (Rakoczy et al.). Then, the paradoxical threat of too much oxygen in the brainstem and the ensuing toxicity, which can result in ictal transgressions is examined (Dean and Stavitzski). Finally, the importance of metabolic deficiencies, with a specific focus on how proliferator-activated receptor gamma coactivator 1-alpha (PGC-1α) activity and mitochondrial development and proliferation, shape the developing fetus and, when insufficient, shapes the development of neural function of breathing is explored (Mohammadi et al.).

Over the years, there has been much debate on the oxygen sensor in the carotid body and how the sensitivity is set for relatively small falls in oxygen levels. Whilst evidence exists for different proposed sensors, only the mitochondria appear to have a unique gene expression signature and phenotype, placing it squarely at the forefront, with the sensitivity being set by the oxygen binding characteristics at complex IV (Bishop and Ratcliffe, 2020). During hypoxia, a decrease in electron transport chain activity and ATP production would be expected. However, there is still much discussion on how this links to the type I cell depolarisation and increased activity seen in hypoxia. In the review by (Holmes et al.), some of the ideas linking electron transport chain activity, reactive oxygen species generation, ATP and MgATP production, metabolic by-product accumulation, and receptor stimulation to elicit carotid body activation are discussed. The rundown of successful electron flow is proposed to generate an increase in ROS from complexes I and II as electrons back up, and mitochondrial ROS then becomes the signal that links the mitochondria to cell membrane depolarization. This occurs in conjunction with a fall in ATP/MgATP generation by mitochondria which can stimulate TASK1/3 and TRPM7 channels. In addition to these concurrent excitatory signals, lactate produced by an increased glycolytic flux has been shown to mediate, in part, the oxygen sensitivity of the carotid bodies. Summarily, (Holmes et al.), describe a likely situation where multiple pathways can impinge on one another or act in concert to elicit the full hypoxic response. This provides a mechanism of specificity regarding the carotid bodies’ response to the full range of oxygen tension encountered in terrestrial life.

At the level of the carotid body type I cell, the role played by mitochondria is clearly important to the carotid bodies’ oxygen sensing capability as discussed by (Rakoczy et al.). They propose a novel oxygen-sensing hypothesis centered on mitochondrial thermal gradients. In essence, the exothermic nature of mitochondrial function allows for a temperature change to influence membrane ion channel activity and thereby depolarise the cell. Whilst there is much work to do on this hypothesis, it is an exciting idea to explore as it relies on signalling microdomains contributing to the carotid bodies’ oxygen sensitivity. This stimulating work raises interesting questions on whether changes in microdomain signalling may be responsible for changes in oxygen sensitivity associated with different diseases.

Hypoxia is commonly associated with detrimental metabolic and neurologic effects. It is, therefore essential to understand the paradoxical events of hyperoxic oxygen toxicity in the brainstem. (Dean and Stavitzski) explore, based on animal studies, the paradoxical situation of hyperoxic hyperventilation. This deleterious situation is a major reason why care and attention need to be paid to hyperbaric therapies. (Dean and Stavitzski) discuss key aspects of the induction of hyperoxic central nervous system stimulation, seizure generation in response to central nervous system hyperoxia toxicity and ROS generation and, practical consideration when comparing *in vitro* brainstem slice preparations treated with hyperoxic 95% oxygen/5% carbon dioxide gas and *in vivo* preparations with normoxic and hypo/poikilocapnic gas. In summary, this important contribution brings to light an often overlooked but, still important, physiologic situation.

Finally, (Mohammadi et al.), synthesize the current knowledge on fetal mitochondrial development and the development of normal breathing. Their review discusses the importance of PGC-1α and its role in mitochondrial development, which is of paramount importance to the developing lung and brain. In their review, they focus on the signalling cascades which activate PGC-1α to explore potential therapies which may stimulate PGC-1α and rectify developmental abnormalities to rescue mitochondrial development and, therefore, lung and central nervous system development to rescue important brainstem breathing centers.

We hope that this special issue of Frontiers in Physiology finds you well and gives intrigue to the regulation of breathing through mitochondrial regulatory control.
